# Horsepower: equine-assisted interventions are associated with reduced PTSD symptoms, improved sleep quality, and quality of life in military veterans with treatment-resistant PTSD

**DOI:** 10.3389/fpsyg.2026.1768001

**Published:** 2026-06-09

**Authors:** Chantal M. Kapteijn, Eric Vermetten, T. Bas Rodenburg, Nienke Endenburg

**Affiliations:** 1 Animals in Science and Society, Department of Population Health Sciences, Faculty of Veterinary Medicine, Utrecht University, Utrecht, Netherlands; 2 Department of Psychiatry, Leiden University Medical Centre, Leiden, Netherlands

**Keywords:** GT9X, heart rate, heart rate variability, non-trauma focused, PCL-5, physiological parameters, psychometric questionnaires

## Abstract

**Introduction:**

Military veterans are at an elevated risk of developing posttraumatic stress disorder (PTSD), while first-line trauma-focused treatments are less effective in this population. This highlights the need for novel, evidence-based complementary interventions for military veterans with treatment-resistant PTSD. Although equine-assisted interventions (EAIs) are increasingly popular and appear promising, many studies suffer from methodological issues. Therefore, further research incorporating both psychometric and physiological parameters is warranted.

**Methods:**

Nine consecutive groups of eight veterans (*n* = 72; 58 men and 14 women) participated in this study. Due to a dropout rate of 8.33% and the exclusion of 11.11% of participants for missing more than two sessions, 58 veterans were included in the dataset. All participants had a prior PTSD diagnosis and/or a score >31 on the PTSD Checklist for DSM-5 (PCL-5) questionnaire at intake and had previous evidence-based treatment but continued to experience symptoms. The program consisted of 12 weekly non-trauma-focused group sessions and one follow-up session (week 24), all lasting from 10:00 to 15:00. Measurements were performed during baseline conditions (B), a 20-min free activity (F) focused on relaxation and social affiliative contact with the horse, and a 30-min directed activity (D) involving caretaking, groundwork, and riding.

**Results:**

Psychometric questionnaires demonstrated a significant decrease in PTSD symptoms (PCL-5), an improvement in sleep quality (Pittsburgh Sleep Quality Index [PSQI]), and an improvement in quality of life (EuroQol 5-Dimension 5-Level questionnaire [EQ-5D-5L]), with effects persisting at least until the 3-month follow-up session. No significant differences in heart rate (HR) or heart rate variability (HRV) were observed between pre- and post-intervention. However, during EAI sessions, patterns consistent with physiological adaptation were observed, as HR decreased and HRV increased after the first week during free interactions with the horse.

**Discussion:**

In conclusion, the EAI program was associated with long-term effects on PTSD, sleep quality, and quality of life in veterans with treatment-resistant PTSD. No structural changes in physiological parameters were found, only evidence for successful adaptation to acute stress during EAI sessions. We hypothesize that adaptation to physiological arousal during acute stress may represent a possible working mechanism of EAI.

## 1 Introduction

The Diagnostic and Statistical Manual of Mental Disorders, Fifth Edition (DSM-5) classifies posttraumatic stress disorder (PTSD) as a mental health condition that develops after witnessing or being exposed to one or more traumatic events (American Psychiatric Association, 2013). PTSD has severe negative consequences for physical and mental health (Asnaani et al., 2014) and is associated with comorbidity such as substance abuse and psychiatric disorders (Goldstein et al., 2016; Sareen et al., 2005; Smith et al., 2016; Thomas et al., 2010), and sleep disturbances (Harvey et al., 2003). Military veterans are a high-risk group for the development of PTSD (Creamer and Forbes, 2004), and its prevalence is greater than that in other population groups (Gates et al., 2012). For Dutch military veterans in the Netherlands, approximately 5–8% suffered from PTSD within 10 years after deployment to Afghanistan, depending on the timing of the measurement (Reijnen et al., 2015; van der Wal et al., 2020). In conclusion, PTSD is a mental health condition that is prevalent and has severe effects on quality of life, indicating the need for effective treatments.

First-line evidence-based treatments for PTSD include trauma-focused therapies (TFTs), such as prolonged exposure (PE), eye movement desensitization and reprocessing (EMDR), and cognitive processing therapy (CPT), with additional pharmacotherapy if needed (Bisson et al., 2007; Charney et al., 2018; Coventry et al., 2020; Watkins et al., 2018). Although generally TFTs are more effective than non-trauma-focused therapies (non-TFTs) (Bisson et al., 2013; Grech and Grech, 2020), they tend to be less effective among military veterans (Coventry et al., 2020; Milligan et al., 2025). A large part of the PTSD patients still experience residual symptoms after treatment (Bradley et al., 2005; Schottenbauer et al., 2008), and 60–72.8% of the military veteran population retained criteria for a PTSD diagnosis for at least one cluster after first-line treatment (Levi et al., 2021; Steenkamp et al., 2015). Poor treatment compliance (Mulick et al., 2011), lower effect sizes (Kitchiner et al., 2019), and high dropout rates (Kitchiner et al., 2019; Mulick et al., 2011; Schottenbauer et al., 2008) may further limit the effectiveness of TFT. Dropout rates are higher for TFT (27.1%) than for non-TFT (16.1%) (Edwards-Stewart et al., 2021). The limited effectiveness of TFT in military veterans with PTSD indicates the need for novel evidence-based complementary (non-TFT) interventions (Starke and Stein, 2017; Steenkamp et al., 2015).

Equine-assisted interactions (EAIs) may be a complementary intervention and have become increasingly popular for patients with mental health issues (Boss et al., 2019; De Santis et al., 2017; Gehrke et al., 2011; Li and Sanchez-Garcia, 2023). EAIs use structured activities involving horses to improve human mental health and wellbeing (De Santis et al., 2017) and biological, physical, psychological, and social functioning (Stern and Chur-hansen, 2020). EAI is an umbrella term that covers both equine-assisted activities and equine-assisted therapies, the latter of which are guided by healthcare professionals (Kinney et al., 2019b; Marchand et al., 2021). Several quantitative studies have shown EAI to have positive effects in military veterans with PTSD (Arnon et al., 2020; Burton et al., 2019; Fisher et al., 2021; Johnson et al., 2018; Lanning et al., 2017; Lanning et al., 2018; Malinowski et al., 2018; Monroe et al., 2019; Romaniuk et al., 2018; Shelef et al., 2019; Steele et al., 2018; Wharton et al., 2019; Willmund et al., 2021; Zhu et al., 2021). However, many suffer from methodological issues such as small sample sizes, a lack of information on dropout rates and/or clear inclusion criteria, a lack of information on the intervention and standardized protocols, and a lack of long-term follow-up or randomized control groups, emphasizing the need for more well-designed studies (Boss et al., 2019; Kapteijn et al., 2025; Kinney et al., 2019a; Li and Sanchez-Garcia, 2023; Marchand et al., 2021; Palomar-Ciria and Bello, 2023). Most quantitative studies on the effects of EAI include primarily psychometric questionnaires, but only a few include psychophysiological parameters (Kapteijn et al., 2025). Although psychometric self-reports are observed as the gold standard for assessing PTSD symptoms, these results can be compromised by memory, emotional, or cultural bias (Yang et al., 2021). This highlights the need for objective additional psychophysiological measures that can assess the effectiveness of EAI.

Psychophysiological measures could serve as objective complementary measures to assess the effectiveness of EAI, as they indicate treatment-related changes in trauma symptoms (Sack et al., 2007; Yang et al., 2021). Heart rate (HR) and heart rate variability (HRV) are promising biomarkers for measuring treatment efficacy in PTSD (Cao-Noya et al., 2025). PTSD is linked to increased HR and reduced HRV (Dennis et al., 2016; Dennis et al., 2014; Hauschildt et al., 2011; Park et al., 2017; Tan et al., 2011), reflecting chronically decreased parasympathetic activity (Hauschildt et al., 2011; Kim et al., 2018), and heightened arousal in response to stress (Dennis et al., 2016). A meta-analysis indicated that HR activity was reduced in response to stressors in PTSD patients after cognitive behavioural therapy (Goncalves et al., 2015). EMDR treatment also led to reduced trauma-script-induced HR responsivity and increased HRV, indicating increased parasympathetic tone and decreases in PTSD symptoms, as assessed by the Impact of Event Scale (IES) questionnaire and the Posttraumatic Stress Diagnostic Scale (PDS) (Sack et al., 2007). There are indications that EAI has favourable effects on HRV by favouring a state of relaxation through activating the parasympathetic nervous system (Garcia-Gomez et al., 2020). Malinowski et al. (2018) found that EAI significantly reduced heart rate on day 2 and PTSD symptoms but had no effect on HRV measures, respiration rate, or blood pressure in military veterans with PTSD. McDuffee et al. (2024) reported both psychological and psychophysiological benefits of EAI for individuals with PTSD; specifically, mood, anxiety, and wellbeing improved, while HR increased and mean RR decreased, which was explained by arousal from a positive stimulus (McDuffee et al., 2024). Evidence regarding the effects of EAI on HR and HRV parameters is inconsistent, which may reflect heterogeneity in the type of activities and relatively small sample sizes, and emphasizes the need for further research.

Studies that include psychophysiological measures are essential for understanding the working mechanisms of interventions involving animals, as they enable the simultaneous investigation of stress-regulatory systems in both humans and animals (Hediger et al., 2019). There are several hypothetical working mechanisms of EAI, such as the human–horse bond, a safe and non-judgmental environment, the experience of control/autonomy, horses as a mirror or metaphor, mindfulness, and nature exposure (Marchand et al., 2021). Commonly described target outcomes of EAI are emotional regulation, relational skills, mindfulness, and attachment/trust building (Bachi et al., 2012; Kendall et al., 2015). However, the exact working mechanisms of EAI are unknown (Marchand, 2023; Marchand et al., 2021).

This study aims to investigate the effects of EAI on military veterans with treatment-resistant PTSD, a population in need of additional treatment options. By integrating psychometric measures with objective psychophysiological measures (HR and HRV), employing a longitudinal design with long-term follow-up, and including a relatively large sample, this study contributes to the existing evidence on the effects of EAI and potential working mechanisms. It is hypothesized that EAI will be associated with decreased PTSD symptoms, as well as improved sleep quality and quality of life, as assessed by validated psychometric questionnaires. At the physiological level, HR and HRV are expected to vary with the type of horse-related activity and to change throughout the program. Specifically, activities that involve affiliative contact and relaxation are hypothesized to be associated with lower HR and higher HRV, whereas more physically demanding activities (e.g., groundwork and riding) are expected to be associated with higher HR and lower HRV. Participating veterans and horses are unfamiliar with one another at the onset of the program, and a subset of the veterans have no prior horse experience. Therefore, we expect significant time effects reflecting the progressive development of the human–horse relationship across the intervention period.

## 2 Materials and method

### 2.1 Subjects

In total, nine consecutive groups of eight veterans (*n* = 72; 58 men, 14 women) volunteered for this study through self-selection. Participants were informed about the program through social media or were referred by their therapist after completing regular treatment. To compensate for seasonal effects, an equal number of groups started during spring and autumn. Participants did not receive compensation; only lunch was provided. All participants were screened by a team that included a psychiatrist, mental healthcare psychologists, and a researcher and were treatment-resistant, defined as experiencing symptoms despite one or more previous evidence-based treatments (Fonzo et al., 2020). The dropout rate was 8.33%, as six individuals dropped out due to personal circumstances. Eight individuals (11.11%) were excluded from the dataset because they were absent for more than two sessions. In total, 58 participants were included in the dataset (for demographic information, see Table 1), each contributing up to 6 PTSD Checklist for DSM-5 (PCL-5), 5 Pittsburgh Sleep Quality Index (PSQI), and 13 EuroQol 5-Dimension 5-Level questionnaire (EQ-5D-5L) repeated measures for psychometric parameters, and 13 baseline, 12 free, and 12 directed repeated measurements for psychophysiological parameters. Inclusion criteria were a PTSD diagnosis according to the participant’s psychological referrer and/or a score >31 on the PCL-5 questionnaire at intake. Exclusion criteria for participation in the program included current addiction, other forms of ongoing therapy during participation, being involved in ongoing legal claims against the Ministry of Defence, and/or aggressive behaviour towards others or animals. Physical impairment was not an exclusion criterion, but veterans had to be able to enter the riding arena with or without aids. A passive hoist was available for mounting the horse if participants’ impairments required it. In total, 37.9% of the participants in the dataset reported current medication use; see Table 1 for more detailed information. Due to variations in dosages and combinations, these medications were analysed collectively, with “current medication use” as a fixed factor rather than separately.

**Table 1:** Demographic characteristics of the participants who completed the program and were included in the dataset.

Characteristic	Included participants
Sex (male/female)	45 (77.6%)/13 (22.4%)
Age at start of the program (mean ± SD)	48.2 ± 10.2
Weight (mean ± SD)	90.5 ± 14.5
Living with a partner (yes/no)	42 (72.4%)/16 (27.6%)
Number of children (mean ± SD)	1.6 ± 1.0
Providing care for children aged < 18 years for ≥ 3 days/week (yes/no)	27 (46.6%)/31 (53.4%)
Experience caring for horses (yes/no)	32 (55.2%)/26 (44.8%)
Experience with horseback riding (yes/no)	39 (67.2%)/19 (32.8%)
Years served (mean ± SD)	14.3 ± 10.8
Number of deployments (mean ± SD)	2.5 ± 2.0
Country of deployment	
Yugoslavia	32 (55.2%)
Afghanistan	30 (51.7%)
Other	17 (29.3%)
Iraq	15 (25.9%)
Lebanon	4 (6.9%)
Comorbidity (past and present)	28 (48.3%)
Depressive disorders	13 (22.4%)
Personality disorders (borderline)	10 (17.2%)
Non-DSM-5 disorders: burnout	9 (15.5%)
Neurodevelopmental disorders (ADHD)	5 (8.6%)
Anxiety disorders	5 (8.6%)
Somatic symptom disorders (conversion disorder, tinnitus)	1 (1.7%)
Substance-related and addictive disorders	4 (6.9%)
Current medication use	22 (37.9%)
SSRIs	11 (19.0%)
Benzodiazepines	8 (13.8%)
Other antidepressants	7 (12.1%)
Other psychoactive medications	8 (13.8%)
SNRIs	1 (1.7%)
TCAs	1 (1.7%)

Years served refers to the number of years employed by the Ministry of Defence and may include civilian positions in addition to active duty. Co-morbidities included both past and present diagnoses.

### 2.2 Equines

Each horse was linked to two veterans per group, and this combination remained stable throughout the entire program. In total, six trained, healthy horses were included in the study (one mare and five geldings, average wither height of 165.58 ± 8.30 cm, average age of 16.17 ± 6.15 y). The horses were housed at a riding school (Stal Groenendaal, Bunschoten, the Netherlands) that also accommodated people with mental and physical disabilities for riding and interacting with horses. The horses were housed in individual boxes with straw bedding, ad libitum hay (fed at 8:00 and 16:00), pellets twice a day (12:00 and 19:30), access to an outside paddock in a group for 1.5–2 h daily and were ridden daily for 1–3 h, in addition to the EAI program.

### 2.3 EAI program

The study was performed from 2020 to 2025 and the EAI program consisted of 12 subsequent, weekly non-trauma-focused group sessions and one follow-up session after a 3-month interval (week 24) all lasting from 10:00 to 15:00. All interactions were performed individually during consecutive turns, in an octagon-shaped ring with a diameter of 11.4 m, demarcated with temporary fencing poles and white ribbons within an indoor riding arena (20 × 40 m). Each day consisted of instructions, a free activity (F: 22.2 min ± 2.8), and a directed activity (D: 30.2 min ± 6.3). Exceptions were week 1, which had no directed activity, and week 24, which had no free activity. During baseline conditions (B), veterans were seated and received instructions for the day within the group for 45 min. During F, veterans focused mainly on making physical contact with the horse and relaxation. From 12:30 to 13:30, veterans had a 1-h lunch break. In the afternoon, veterans had a specific assignment together with the horse (D), including both unmounted and mounted activities such as caring for the horse, groundwork, and riding. For more detailed information on the program’s directed activities, see Table 2. The interventions were delivered by a mental healthcare psychologist and a behavioural biologist specialized in horse behaviour, under the guidance of a psychiatrist and a psychologist specialized in PTSD in military veterans. This core team of professionals was present across all groups to deliver interventions in accordance with a standardized protocol, ensuring treatment fidelity. Each day, the program ended with a 45-min group evaluation and reflection.

**Table 2:** Overview of the EAI program by day.

Type	Day	Activity	Psychometric questionnaires	Location
Caretaking	1	—	PCL-5, EQ-5D-5L, PSQI	Ring
		Free activity		Ring
	2	Free activity	EQ-5D-5L	Ring
		Brushing the horse		Ring
	3	Free activity	EQ-5D-5L	Ring
		Haltering and leading the horse through an S-shaped form of beams		Ring
	4	Free activity	PCL-5, EQ-5D-5L, PSQI	Ring
		Lunging the horse (including trotting)		Ring
Groundwork	5	Free activity	EQ-5D-5L	Ring
		Leading the horse around cones in a figure eight		Ring
	6	Free activity	EQ-5D-5L	Ring
		Leading the horse to step over three cavaletti		Ring
	7	Free activity	EQ-5D-5L	Ring
		Leading the horse over a plastic cover		Ring
	8	Free activity	PCL-5, EQ-5D-5L, PSQI	Ring
		Leading the horse through an obstacle course		Riding arena
Riding	9	Free activity	EQ-5D-5L	Ring
		Riding on a bareback pad, guided by hand		Ring
	10	Free activity	EQ-5D-5L	Ring
		Riding with a saddle and bridle, on a lunge line		Ring
	11	Free activity	EQ-5D-5L	Ring
		Riding with a saddle and bridle, loose withing the ring		Ring
	12	Free activity	PCL-5, EQ-5D-5L, PSQI	Ring
		Riding with a saddle and bridle		Riding arena
	24	Riding with a saddle and bridle	PCL-5, EQ-5D-5L, PSQI	Riding arena

The first row for each day presents the morning activity and the second row presents the afternoon activity, including the questionnaires completed and the activity location.

### 2.4 Psychometric measures

Outcome assessment was conducted using standardized, psychometrically validated instruments commonly used in trauma and mental health research. The PCL-5 is a 20-item self-rating Likert scale, with scores ranging from 0 to 80, that assesses the DSM-5 criteria symptoms of PTSD with excellent psychometric properties, as it has good internal consistency, test–retest reliability, and convergent and discriminant validity (Blevins et al., 2015; Bovin et al., 2016; Forkus et al., 2023; Wortmann et al., 2016). The cut-off score is 31–33 for diagnosing PTSD (Bovin et al., 2016; Forkus et al., 2023; Murphy et al., 2017), and factor analysis revealed that data were best explained by a 7-factor hybrid model (Armour et al., 2015; Blevins et al., 2015; Bovin et al., 2016; Forkus et al., 2023; Wortmann et al., 2016). The Dutch version of the PCL-5 was used in this study and is validated for Dutch trauma-exposed adults (Hoeboer et al., 2024; Van Praag et al., 2020).

The Pittsburgh Sleep Quality Index (PSQI) is a 19-item self-report measure that assesses sleep quality and disturbance over the past month with good internal consistency, reliability, and construct validity in a variety of clinical populations (Buysse et al., 1988; Carpenter and Andrykowski, 1998; Mollayeva et al., 2016), including military veterans (Insana et al., 2013). The 19-item measure generates seven component scores that can be summed to form a single global score (0–21), with a PSQI score of > 5 distinguishing poor sleepers from good sleepers, with high diagnostic sensitivity and specificity (Buysse et al., 1988). However, in military veterans, a cut-off score of ≥ 4 could accurately identify those who met diagnostic criteria for PTSD (Insana et al., 2013). The factor structure of sleep quality varies across populations, but the global score can be used to quantify sleep quality (Mollayeva et al., 2016).

The EQ-5D-5L is a self-report measure that assesses health-related quality of life (HRQoL) and has excellent psychometric properties across a broad range of populations (Feng et al., 2021) and good discriminative ability, test–retest reliability, and construct validity in patients with PTSD (Dams et al., 2021). It consists of five dimensions: mobility, self-care, usual activities, pain/discomfort, and anxiety/depression that can be converted into a health utility measure using country-specific weights and a Visual Analogue Scale (VAS) (Feng et al., 2021). Answers for the dimensions are given on five level scale with “no problems (1),” “slight problems (2),” “moderate problems (3),” “severe problems (4),” or “extreme problems (5)” which can be combined to generate 5^5^ possible health profiles, with “11111” and “55555” representing the best and worst health state, respectively (Dams et al., 2021; Euroqol Research Foundation, 2025). The health profiles are combined into a single measure of health utility, a summary index score (EQ-5D index), using preference-based value sets derived from the Dutch general population that provide weights to each health state profile (Euroqol Research Foundation, 2025; Versteegh et al., 2016). Index scores generally range from 0 (a health state equivalent to death, with negative values representing health states worse than death) to 1 (full health) (Dams et al., 2021; Feng et al., 2021; Euroqol Research Foundation, 2025). In addition, using the EuroQol Visual Analogue Scale (EQ-VAS), participants rated their HRQoL visually on a scale from 0 (worst) to 100 (best) (Adams et al., 2015; Euroqol Research Foundation, 2025). The EQ-5D index is a value attached to an EQ-5D profile using a set of weights that reflect on average people’s preferences for how good or bad a health state is. The EQ-VAS represents the patients’ perspective of their health (Euroqol Research Foundation, 2025).

### 2.5 HR and HRV

Psychophysiological measurements were obtained using a wearable, non-invasive HR monitor to assess autonomic activation during baseline (B) and task-related (F and D) conditions. In addition, HR and HRV were measured in the veterans’ home environments during the week before the program (pre) and the week after the program (post). Veterans were given at least 5 min to adapt to wearing the elastic girth and equipment before baseline measurements started. Baseline recordings lasted for 5 min while veterans were sitting and receiving a verbal explanation of the program for that day. Recordings during F lasted at least 15 min and during directed activities at least 30 min, except for the obstacle course, which lasted at least 2 min. Polar H10 Heart Rate Sensor transmitters and Polar M430 receivers (Polar Electro Nederland, Utrecht, The Netherlands) were used to obtain interbeat interval (IBI) recordings, as portable devices are both convenient and provide accurate results compared with ECG (Dobbs et al., 2019), even in non-stationary conditions (Gilgen-Ammann et al., 2019). Polar H10 is a suitable wearable for military populations in a field setting (Hinde et al., 2021). All raw IBI data were analysed with software (Kubios HRV, University of Eastern Finland, Kuopio Finland) for HR (beats per minute) and HRV (milliseconds) parameter analysis (Tarvainen et al., 2014), after applying the strong correction filter, which is suitable for adults (Alcantara et al., 2020; Aranda et al., 2017). Measurements with a percentage of beats correction ≥ 10% were excluded from the analysis as these may affect HRV parameters (Kristiansen, 2011; Malik et al., 1996; Peltola, 2012; Rogers et al., 2021). The time-domain parameter root mean square of successive differences (RMSSD) was analysed, as it estimates short-term components of HRV (Malik et al., 1996; Tarvainen et al., 2014; von Borell et al., 2007) and reflects the parasympathetic activity within the autonomic nervous system (Baevsky and Chernikova, 2017). The RMSSD also seems to be one of the most sensitive parameters of HRV when used as a biomarker of PTSD treatment efficacy (Cao-Noya et al., 2025). A systematic review indicates that HRV is significantly reduced during mental stress, with a shift in the autonomic nervous system towards sympathetic activation and parasympathetic withdrawal (Castaldo et al., 2015; Delaney and Brodie, 2000).

### 2.6 Activity

Objective physical activity metrics were obtained using a triaxial accelerometer, allowing quantification of movement. Physical activity data of the veterans were accurately measured by the ActiGraph accelerometer (GT9X) using a 15-s epoch (Evenson et al., 2015; Powell et al., 2017), which was worn on the hip (Evenson et al., 2015; Hwang et al., 2018; Powell et al., 2017). Raw data were analysed in ActiLife (version 6.13.6, ActiGraph, Florida, USA). The variable vector magnitude count per minute (VMCPM) is a single parameter that combines movement from three axes, providing a robust measure of physical activity intensity per minute. Each axis produces counts, representing acceleration during a time window (epoch), and these are combined into one value: vector magnitude (VM) = √ (X^2^ + Y^2^ + Z^2^).

### 2.7 Statistical analysis

Before the start of the study, a power analysis was performed and approved by the Medical Ethical Committee (NL75890.041.20). Analyses were conducted using linear mixed models, with model specifications tailored to each outcome variable to ensure optimal fit and statistical validity. For each measure separately, the covariance structure was based on the Akaike’s Information Criterion (AIC) index, provided it was mathematically stable and reached convergence (Chan, 2004; Duricki et al., 2016), and this is reported in the Results section. For all measures, the individual ID was incorporated as a random factor with the intercept included; week was the repeated factor; number of deployments was a covariate; and sex, current medication use, experience with horses, and group were fixed factors. Interaction effects between week and the other factors were also included, except for week * group, as this negatively affected convergence due to the many levels and did not significantly improve model fit. Group was modelled as a fixed effect in all analyses, rather than estimating a group-level random effect, which is unstable with so few clusters and leads to convergence issues. Model selection was performed using stepwise backward deletion (Chan, 2004; Duricki et al., 2016) of non-significant factors based on a reduction in AIC of>2 points (Burnham and Anderson, 2004). If factors were deleted, this is reported in the Results section. The repeated factor week was always retained in the model because it was of primary interest. The residuals for all clusters were normally distributed according to the (exact) Kolmogorov–Smirnov one-sample test. If residuals were not normally distributed, a log_10_ transformation was applied, and the QQ plot was visually inspected for normality; small deviations in the tails and biologically relevant outliers were considered acceptable. For the overall fixed effects, effect sizes were estimated from the mixed-model test statistics (Lakens, 2013). Partial eta squared ( $\eta_{p}^{2})$ was calculated from the F statistic of the fixed effects as an approximation of the magnitude of the predictor ( $\eta_{p}^{2}=\frac{F\times df_{\text{effect}}}{F\times df_{\text{effect}}+df_{\text{error}}}$ ), this quantifies how much variance in the dependent variable is explained by a specific predictor (fixed effect) after accounting for other predictors in the model.

## 3 Results

### 3.1 Psychometric questionnaires

#### 3.1.1 PCL-5

**Figure 1:**
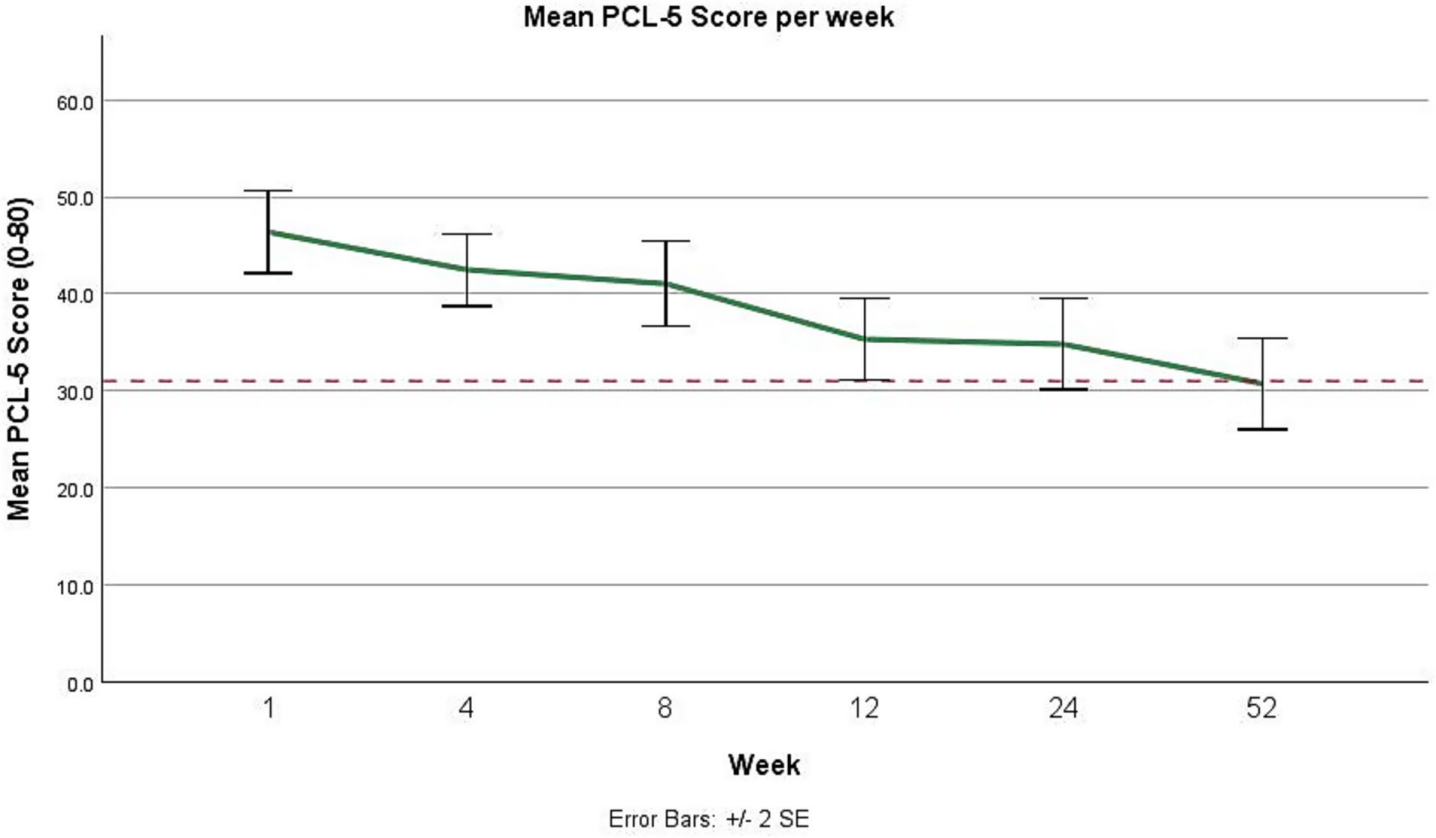
Mean PCL-5 scores over time with standard errors (SEs). The dotted red line indicates the PTSD cut-off score of 31.

PCL-5 scores significantly decreased over time (*F*(5, 73.03) = 8.62, *p* < 0.001, η^2^p = 0.371, indicating a large effect with 37.1% of the variance explained); see Figure 1 for raw values. Residuals were normally distributed, and removal of non-significant fixed effects did not improve model fit (AR1 heterogeneous) based on AIC. *Post-hoc* comparisons showed that PCL-5 scores differed across most weeks, with a few exceptions. Specifically, no differences were observed between weeks 4 and 8, nor among weeks 12, 24, and 52. These findings suggest that effects persist beyond the intervention period (see Table 3 for estimated marginal means). Experience with horses (*F*(1, 42.92) = 4.10, *p* = 0.049, η^2^p = 0.087, indicating a moderate effect with 8.7% of the variance explained) significantly affected the PCL-5 score. Participants with prior horse experience reported higher PCL-5 scores (estimated marginal means ± SE: 42.578 ± 2.754) than those without prior experience (estimated marginal means ± SE: 34.907 ± 3.270). No main effects of group, sex, medication, or number of deployments, and no significant interaction effects, were found (see Supplementary Table A). This indicates that the decrease in PCL-5 score over the weeks did not depend on the participants’ sex, medication use, experience with horses, or number of deployments.

**Table 3:** Estimated marginal means of PCL-5 scores over time with standard errors (SEs) and 95% confidence intervals (CIs).

Week	Model estimate	SE	95% CI (lower)	95% CI (upper)
1	46.670	2.623	41.428	51.911
4	42.505	2.434	37.602	47.407
8	41.632	2.474	36.672	46.591
12	35.362	2.550	30.247	40.477
24	34.449	2.754	28.956	39.942
52	31.838	2.757	26.324	37.352

#### 3.1.2 Sleep quality (PSQI)

**Figure 2:**
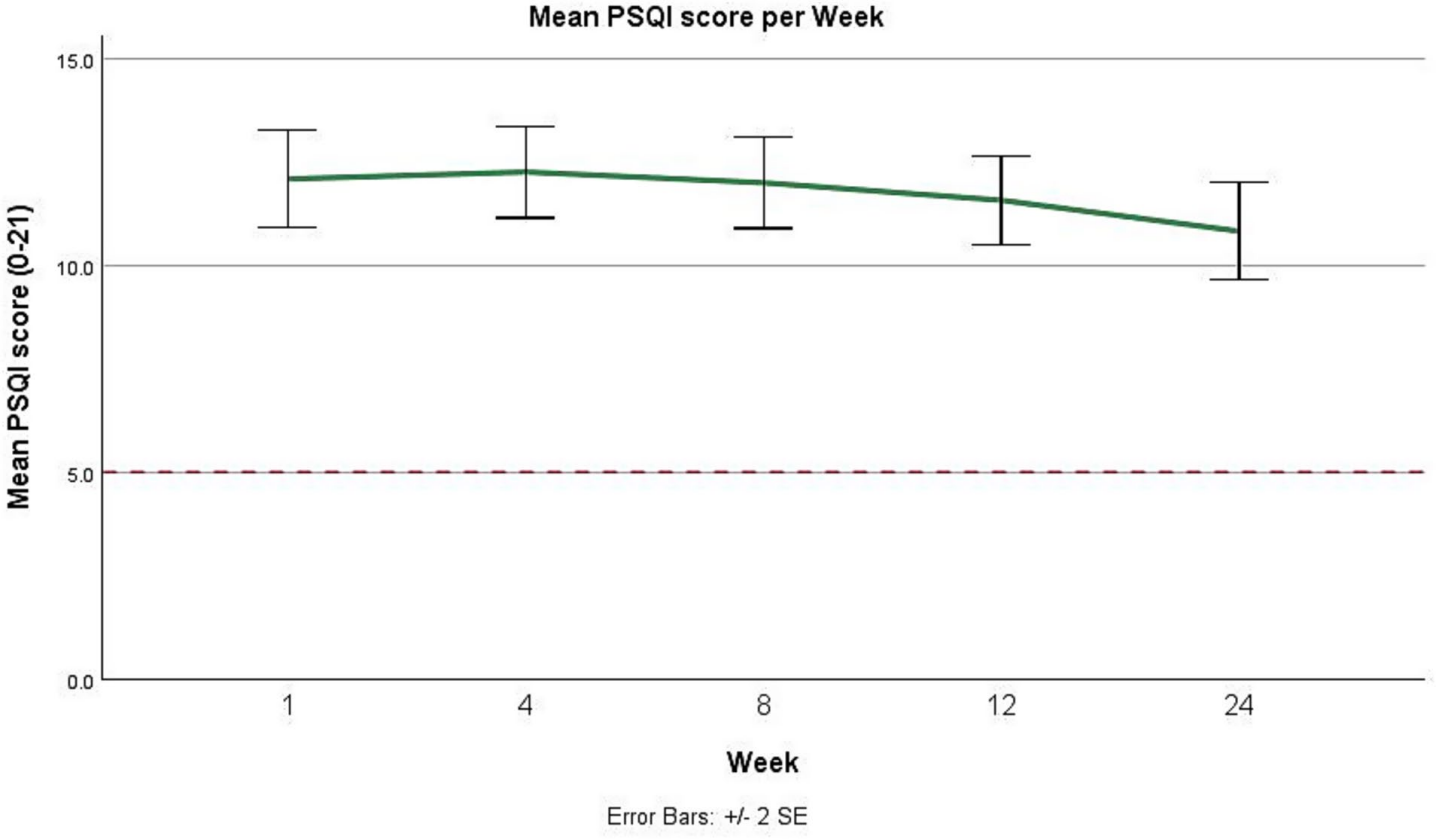
Mean PSQI scores over time with the standard errors (SEs). The dotted red line indicates the cut-off score for poor sleepers.

PSQI scores decreased significantly over time (*F* (4, 55.15) = 5.96, *p* < 0.001, η^2^p = 0.302, indicating a large effect with approximately 30.2% of the variance explained); see Figure 2 for raw values. Residuals were normally distributed, and removal of non-significant fixed effects did not improve model fit based on AIC, with a diagonal covariance structure providing the best fit. Estimated marginal means indicated a gradual decrease in PSQI scores over time, suggesting an overall improvement in sleep quality. Reductions were modest during the initial weeks, with more pronounced reductions observed after week 8 (see Table 4 for estimated marginal means). This pattern suggests that improvements in sleep quality became evident during later phases of the intervention. No significant main effects were found for group, sex, medication, experience with horses, or number of deployments, nor were any significant interaction effects observed (see Supplementary Table B). This indicates that the improvement in sleep quality over the weeks did not depend on the participants’ sex, medication use, experience with horses, or number of deployments.

**Table 4:** Estimated marginal means of PSQI scores over time with standard errors (SEs) and 95% confidence intervals (CIs).

Week	Model estimate	SE	95% CI (lower)	95% CI (upper)
1	12.709	0.750	11.212	14.206
4	12.413	0.695	11.020	13.806
8	12.449	0.686	11.073	13.825
12	11.338	0.719	9.900	12.777
24	10.632	0.859	8.921	12.342

#### 3.1.3 Quality of life (EQ-5D-5L)

**Figure 3:**
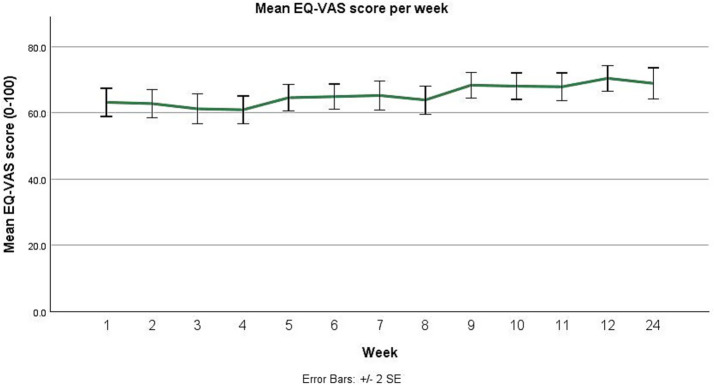
Mean EQ-VAS scor over time with the standard errors (SEs).

The EQ-VAS score increased significantly over time (*F* (12, 295.28) = 2.33, *p* = 0.007, η^2^p = 0.086, indicating a medium effect with approximately 8.6% of the variance explained); see Figure 3 for raw values. Residuals were normally distributed, and removal of non-significant fixed effects did not improve model fit based on AIC, with an ARMA(1,1) covariance structure providing the best fit. *Post-hoc* comparisons showed an overall gradual increase, with confidence intervals overlapping across adjacent time points and later weeks showing higher values (see Table 5 for model estimates). No main effects of group, sex, medication, experience with horses, or number of deployments, nor interaction effects, were found (see Supplementary Table C). This indicates that the improvement in self-perceived health over the weeks was not moderated by the participants’ sex, medication use, experience with horses, or number of deployments.

**Table 5:** Estimated marginal means of EQ-VAS scores over time with standard errors (SEs) and 95% confidence intervals (CIs).

Week	Model estimate	SE	95% CI (lower)	95% CI (upper)
1	62.997	2.718	57.581	68.413
2	61.748	2.712	56.341	67.154
3	61.056	2.715	55.644	66.468
4	61.913	2.717	56.497	67.329
5	63.317	2.735	57.868	68.766
6	64.625	2.757	59.137	70.114
7	65.690	2.718	60.273	71.107
8	63.758	2.720	58.338	69.178
9	66.785	2.730	61.346	72.225
10	68.303	2.720	62.882	73.724
11	66.999	2.728	61.564	72.434
12	70.624	2.712	65.217	76.030
24	69.614	2.720	64.192	75.035

### 3.2 Pre–post comparisons

#### 3.2.1 Heart rate

The HR did not differ significantly between pre- and post-intervention assessments (*F* (1, 39.84) = 2.33, *p* = 0.135). Confidence intervals were relatively wide and substantially overlapped, reflecting a lack of statistical evidence for a change in HR, rather than strong evidence for the absence of an effect. Residuals were normally distributed, and removal of non-significant fixed effects did not improve model fit based on AIC, with a diagonal covariance structure providing the best fit. No main effects of group, sex, medication, experience with horses, or number of deployments, nor interaction effects with week, were found (see Supplementary Table D).

#### 3.2.2 Heart rate variability (RMSSD)

The RMSSD also did not differ significantly between pre- and post-intervention assessments (*F* (1, 41.56) = 0.39, *p* = 0.535). Confidence intervals were relatively wide and substantially overlapped, reflecting a lack of statistical evidence for a change in HRV, rather than strong evidence for the absence of an effect. Residuals were normally distributed, and removal of non-significant fixed effects did not improve model fit based on AIC, with a scaled dentity covariance structure providing the best fit. Medication significantly affected the RMSSD in the pre–post comparison (*F* (1, 36.46) = 4.94, *p* = 0.033, η^2^p = 0.119 indicating a large effect with approximately 11.9% of the variance explained), with participants using medication demonstrating lower RMSSD values (estimated marginal means ± SE: 21.012 ± 2.596) than participants who did not use medication (estimated marginal means ± SE: 27.813 ± 2.205). This suggests reduced HRV among participants using medication. No main effects of sex, experience with horses, or number of deployments, nor interaction effects, were found (see Supplementary Table E).

### 3.3 Baseline

#### 3.3.1 Heart rate

**Figure 4:**
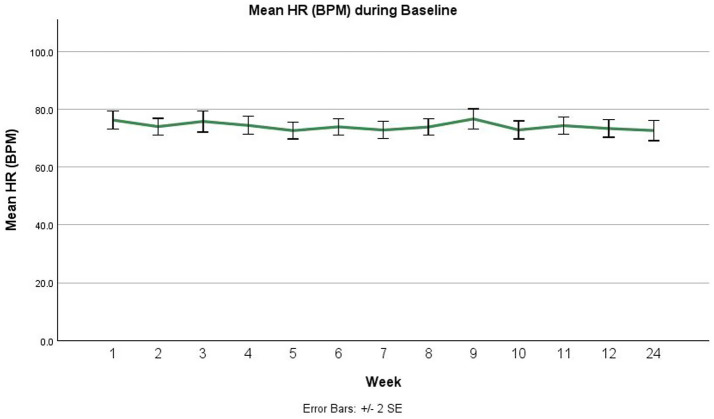
Mean heart rate (HR), expressed in beats per minute (BPM), during baseline conditions with standard errors (SEs).

Baseline HR values were log_10_-transformed to improve the normality of the residuals. Model fit improved when fixed effects of group, deployments, experience with horses, and sex, as well as the interaction effects with week, were removed from the final AR(1) heterogeneous model. During baseline conditions, HR differed significantly over time (*F* (12, 100.51) = 2.32, *p* = 0.012, η^2^p = 0.217 indicating a large effect with approximately 21.7% of the variance explained); see Figure 4 for raw values. *Post-hoc* comparisons indicated that log_10_-transformed HR was primarily higher in weeks 1 and 9 than in most other weeks (see Table 6 for model estimates). Medication use had a significant effect on log_10_-transformed HR (*F* (1, 55.79) = 5.73, *p* = 0.020, η^2^p = 0.093 indicating medium effect size with approximately 9.3% of the variance explained), with higher values observed in participants using medication (estimated marginal means ± SE: 1.887 ± 0.012) than in those who did not use medication (estimated marginal means ± SE: 1.851 ± 0.009). The confidence intervals showed minimal overlap, supporting the robustness of this difference. This indicates that participants who use medication have higher HR values during baseline conditions.

**Table 6:** Estimated marginal means of log_10_-transformed mean heart rate (HR), expressed in beats per minute (BPM), during baseline conditions with standard errors (SEs) and 95% confidence intervals (CIs).

Week	Estimated marginal means	SE	95% CI (lower)	95% CI (upper)
1	1.882	0.010	1.863	1.901
2	1.869	0.008	1.852	1.886
3	1.878	0.011	1.857	1.899
4	1.871	0.008	1.854	1.888
5	1.863	0.008	1.846	1.879
6	1.868	0.008	1.851	1.884
7	1.864	0.008	1.848	1.881
8	1.869	0.008	1.852	1.885
9	1.882	0.009	1.865	1.900
10	1.859	0.009	1.842	1.876
11	1.870	0.009	1.853	1.887
12	1.862	0.009	1.844	1.879
24	1.862	0.008	1.844	1.878

#### 3.3.2 Heart rate variability (RMSSD)

Baseline RMSSD values were log_10_-transformed to improve normality of residuals. Model fit improved when fixed effects of group, sex, number of deployments, as well as interaction effects with week, were removed from the final AR(1) model. During baseline conditions, log_10_-transformed RMSSD did not differ significantly over time (*F* (12, 336.06) = 1.68, *p* = 0.070). Medication use had a significant effect on log_10_-transformed RMSSD (*F* (1, 54.95) = 13.36, *p* < 0.001, η^2^p = 0.196 indicating a large effect size with approximately 19.6% of the variance explained), with lower values observed in participants using medication (estimated marginal means ± SE: 1.263 ± 0.032) than in those who did not use medication (estimated marginal means ± SE: 1.410 ± 0.025). Experience with horses had a significant effect on log_10_-transformed RMSSD (*F* (1, 55.05) = 6.89, *p* = 0.011, η^2^p = 0.111 indicating a medium effect size with approximately 11.1% of the variance explained), with lower values observed in participants with experience with horses (estimated marginal means ± SE: 1.285 ± 0.027) than in those without experience (estimated marginal means ± SE: 1.388 ± 0.030). These results indicate that participants who use medication and/or have experience with horses have lower HRV, indicative of reduced parasympathetic activity.

### 3.4 Free interactions

#### 3.4.1 Heart rate

**Figure 5:**
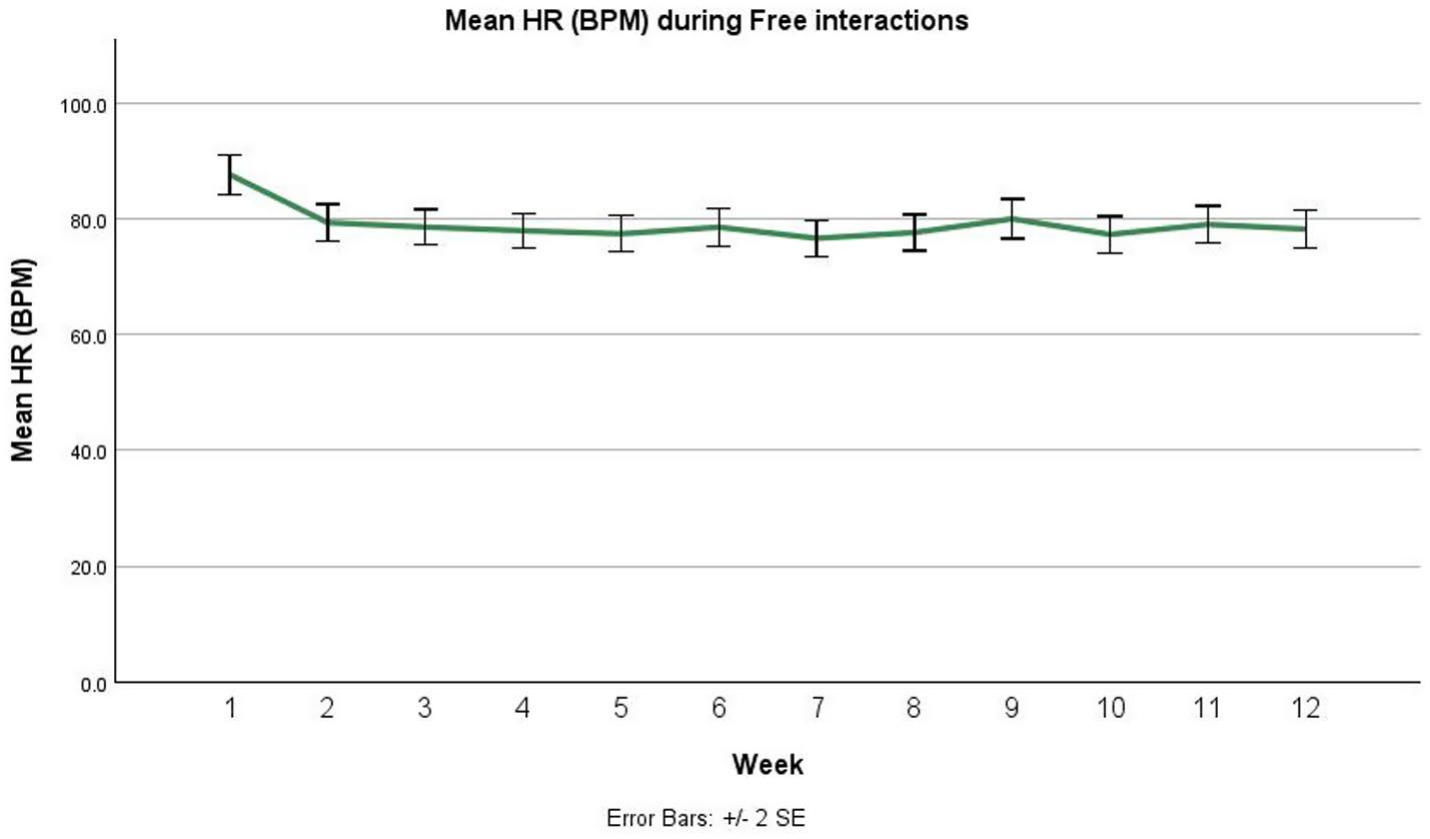
Mean heart rate (HR) expressed in beats per minute (BPM) during free interactions with standard errors (SEs).

HR values during free interactions were log_10_-transformed to improve the normality of the residuals. Model fit improved when fixed effects of group, sex, medication, experience with horses, number of deployments, and interaction effects with week were removed from the model (first-order ante-dependence). A significant difference in log_10_-transformed HR over time was found (*F* (11, 73.22) = 7.63, *p* < 0.001, η^2^p = 0.534 indicating a large effect size with approximately 53.4% of the variance explained); see Figure 5 for raw values. *Post-hoc* comparisons indicated that HR was primarily elevated in weeks 1 and 9 compared with other weeks (see Table 7 for model estimates).

**Table 7:** Estimated marginal means of log_10_-trasnformed mean heart rate, expressed in beats per minute (BPM), during free interactions with standard errors (SEs) and 95% confidence intervals (CIs).

Week	Model estimates	SE	95% CI (lower)	95% CI (upper)
1	1.938	0.010	1.918	1.958
2	1.891	0.009	1.874	1.909
3	1.889	0.009	1.872	1.906
4	1.884	0.009	1.867	1.901
5	1.887	0.009	1.869	1.904
6	1.889	0.009	1.872	1.906
7	1.883	0.008	1.886	1.900
8	1.886	0.008	1.870	1.902
9	1.897	0.009	1.880	1.914
10	1.882	0.009	1.864	1.900
11	1.894	0.009	1.876	1.913
12	1.885	0.009	1.866	1.903

#### 3.4.2 Heart rate variability (RMSSD)

**Figure 6:**
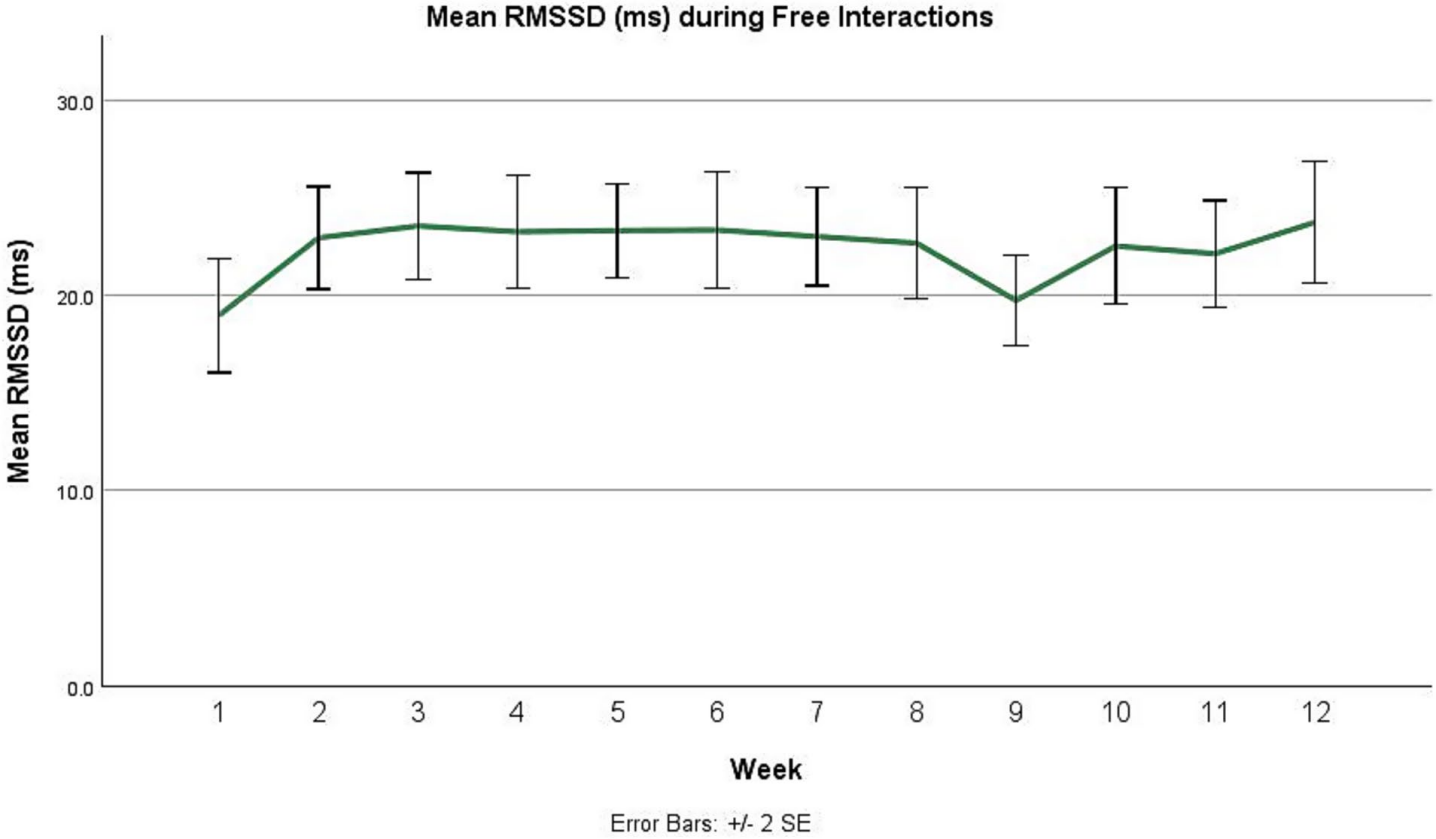
Mean heart rate variability (RMSSD), expressed in milliseconds (ms), during free interactions with standard errors (SEs).

The RMSSD values were log_10_-transformed, and the model (first-order ante-dependence) improved when the main factor group, sex, the number of deployments, and the interaction effects with week were removed. The final model showed a significant change over time in log_10_-transformed RMSSD (*F* (11, 73.84) = 5.02, *p* < 0.001, η^2^p = 0.428, indicating a large effect size with 42.8% of the variance explained); see Figure 6 for raw values. *Post-hoc* comparisons revealed that log_10_-transformed RMSSD was lowest in week 1 and week 9 (see Table 8 for model estimates), indicative of lower parasympathetic activity. Medication significantly affected the log_10_ transformed RMSSD (*F* (1, 54.91) = 7.21, *p* = 0.010, η^2^p = 0.116 indicating a medium effect size with 11.6% of the variance explained), with lower values observed in participants using medication (estimated marginal means ± SE: 1.248 ± 0.032) than in those who did not use medication (estimated marginal means ± SE: 1.356 ± 0.025). Experience with horses significantly affected the log_10_ transformed RMSSD over time (*F* (1, 54.89) = 4.85, *p* = 0.032, η^2^p = 0.081, indicating a medium effect size with 8.1% of the variance explained), with lower values observed in participants who had prior experience with horses (estimated marginal means ± SE: 1.258 ± 0.027) than in those without prior experience (estimated marginal means ± SE: 1.345 ± 0.030). These results indicate that participants who use medication and/or have experience with horses have lower HRV, indicative of decreased parasympathetic activity.

**Table 8:** Estimated marginal means of log_10_-transformed heart rate variability (RMSSD), expressed in milliseconds (ms), during free interactions with standard errors (SEs) and 95% confidence intervals (CIs).

Week	Model estimates	SE	95% CI (lower)	95% CI (upper)
1	1.203	0.033	1.137	1.268
2	1.319	0.023	1.274	1.364
3	1.323	0.026	1.272	1.374
4	1.322	0.025	1.273	1.371
5	1.326	0.024	1.279	1.374
6	1.318	0.026	1.267	1.369
7	1.322	0.022	1.277	1.367
8	1.308	0.023	1.263	1.353
9	1.249	0.024	1.201	1.297
10	1.310	0.026	1.259	1.362
11	1.290	0.026	1.238	1.341
12	1.330	0.025	1.280	1.381

#### 3.4.3 Vector magnitude (counts per minute)

The vector magnitude (VM) values during free interactions were log_10_-transformed to improve the normality of residuals. Model fit improved when fixed effects of group, number of deployments, experience with horses, and interaction effects, with the exception of week * sex, were removed from the final model (toeplitz). During free interactions, the log_10_-transformed VM changed significantly over time (*F* (11, 150.90 = 2.24, *p* = 0.015, η^2^p = 0.140 indicating a large effect size with 14.0% of the variance explained). This temporal pattern was further qualified by a week * sex interaction effect (*F* (11, 150.90) = 2.12, *p* = 0.022, η^2^p = 0.134, indicating a medium effect size with 13.4% of the variance explained), indicating that changes in VM across weeks differed between men and women. However, both the main effect of week and the interaction effect were sensitive to the choice of covariance structure, suggesting that these results are not robust and should be interpreted with caution. Estimated marginal means indicated small and inconsistent sex differences across weeks, with overlapping confidence intervals (see Table 9). No significant effect of sex was found (*F* (1, 60.98) = 0.28, *p* = 0.596). Medication significantly affected the log_10_-transformed VM (*F* (1, 53.03) = 7.68, *p* = 0.008, η^2^p = 0.127 indicating a medium effect size with 12.7% of the variance explained), with higher values observed in participants using medication (estimated marginal means ± SE: 2.658 ± 0.052) than in those not using medication (estimated marginal means ± SE: 2.493 ± 0.041).

**Table 9:** Estimated marginal means of log_10_-transformed vector magnitude (VM), expressed in counts per minute (CPM) per sex, during free interactions with standard errors (SEs) and 95% confidence intervals (CIs).

Week	Females				Males			
	Model estimates	SE	95% CI (lower)	95% CI (upper)	Model estimates	SE	95% CI (lower)	95% CI (upper)
1	2.721	0.094	2.535	2.906	2.666	0.049	2.570	2.762
2	2.531	0.090	2.353	2.708	2.665	0.048	2.570	2.761
3	2.487	0.090	2.309	2.664	2.604	0.049	2.509	2.700
4	2.443	0.090	2.265	2.620	2.610	0.049	2.515	2.706
5	2.664	0.094	2.479	2.849	2.579	0.049	2.482	2.676
6	2.444	0.094	2.259	2.629	2.657	0.049	2.561	2.753
7	2.427	0.092	2.246	2.608	2.548	0.050	2.450	2.645
8	2.549	0.090	2.372	2.727	2.576	0.049	2.479	2.673
9	2.557	0.092	2.376	2.738	2.536	0.049	2.440	2.633
10	2.624	0.092	2.443	2.805	2.502	0.050	2.405	2.600
11	2.613	0.092	2.432	2.764	2.611	0.049	2.515	2.707
12	2.616	0.090	2.439	2.794	2.576	0.049	2.480	2.673

### 3.5 Directed interactions

#### 3.5.1 Heart rate

**Figure 7:**
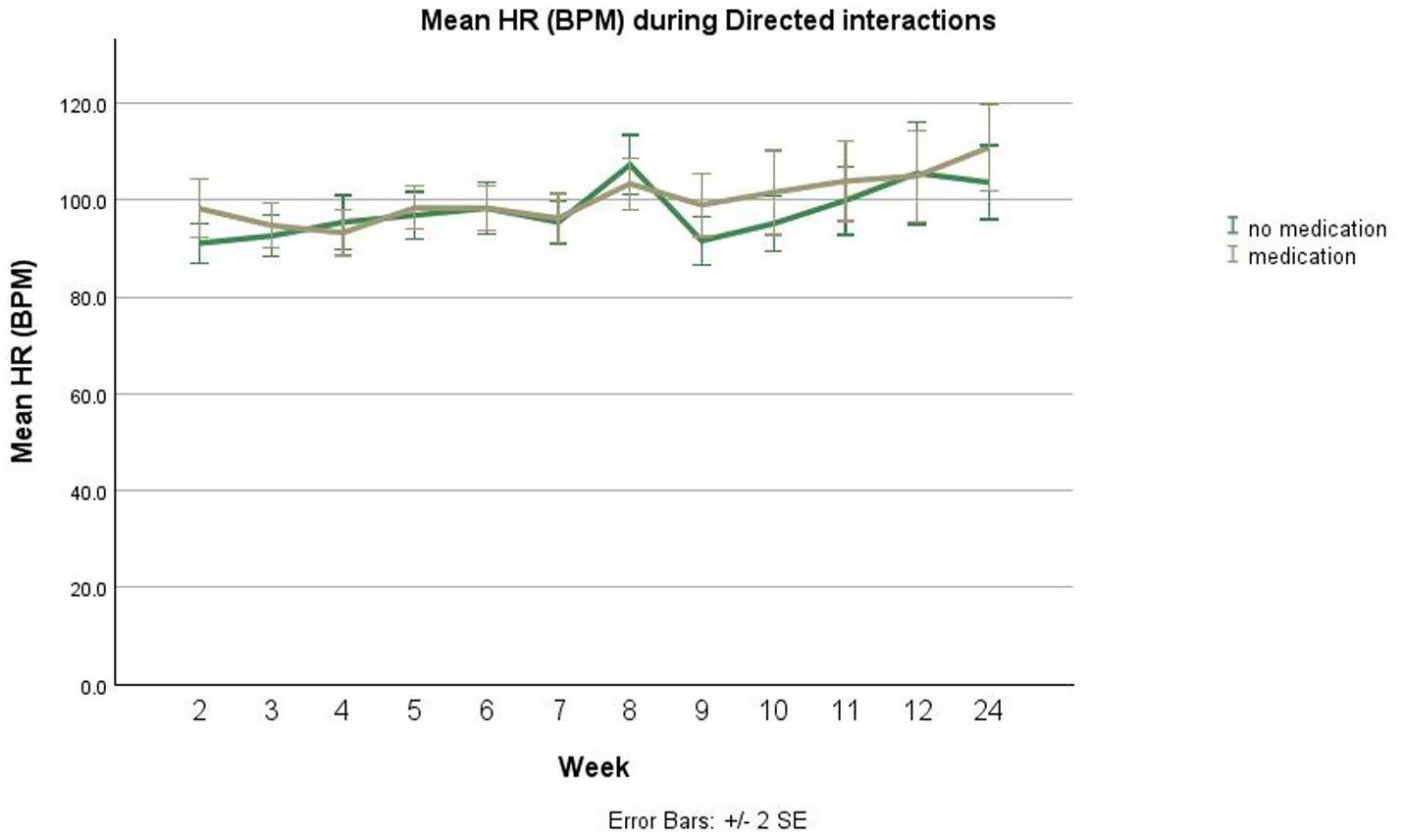
Mean heart rate (HR), expressed in beats per minute (BPM), by medication group during directed interactions with standard errors (SEs).

HR values during directed interactions were log_10_-transformed to improve the normality of the residuals. Model fit for the AR(1) heterogeneous covariance structure improved when fixed effects of group, number of deployments, experience with horses, sex, and interaction effects, with the exception of week * medication, were removed from the final model. A significant effect of time was observed (*F* (11, 94.61) = 12.07, *p* < 0.001, η^2^p = 0.584, indicating a large effect size with 58.4% of the variance explained); see Figure 7 for raw values. This indicates that the log_10_-transformed HR values differed significantly over time. No main effect of medication was found (*F* (1, 70.66) = 1.02, *p* = 0.316). However, there was a significant interaction effect of week * medication use (*F* (11, 94.61) = 2.47, *p* = 0.009, η^2^p = 0.223, indicating a large effect size with 22.3% of the variance explained), indicating that the effect of medication on HR differed across time points. *Post-hoc* comparisons revealed that participants using medication generally exhibited higher HR than those not using medication.

However, the magnitude of this difference differed across weeks and even reversed at weeks 4 and 8 (see Table 10 for model estimates). Inspection of temporal patterns indicated that log_10_-transformed HR values were high in specific weeks involving greater physical exercise, such as the obstacle course (week 8) and riding activities (week 24).

**Table 10:** Estimated marginal means of mean heart rate (HR), expressed in beats per minute (BPM), by medication group during directed interactions with standard errors (SEs) and 95% confidence intervals (CIs).

Week	No medication				Medication			
	Model estimates	SE	95% CI (lower)	95% CI (upper)	Model estimates	SE	95% CI (lower)	95% CI (upper)
2	1.956	0.011	1.933	1.978	1.989	0.015	1.959	2.018
3	1.962	0.010	1.941	1.982	1.972	0.013	1.945	1.999
4	1.973	0.010	1.952	1.993	1.964	0.013	1.938	1.990
5	1.983	0.011	1.961	2.005	1.992	0.014	1.963	2.020
6	1.987	0.011	1.966	2.008	1.989	0.014	1.962	2.016
7	1.971	0.010	1.951	1.991	1.981	0.013	1.956	2.007
8	2.024	0.012	2.001	2.047	2.006	0.015	1.977	2.035
9	1.957	0.010	1.936	1.978	1.992	0.013	1.966	2.019
10	1.973	0.011	1.950	1.996	2.000	0.015	1.971	2.029
11	1.989	0.012	1.966	2.013	2.011	0.015	1.981	2.041
12	1.967	0.039	1.888	2.046	2.017	0.050	1.917	2.116
24	2.017	0.011	1.995	2.040	2.037	0.015	2.008	2.067

#### 3.5.2 Heart rate variability (RMSSD)

**Figure 8:**
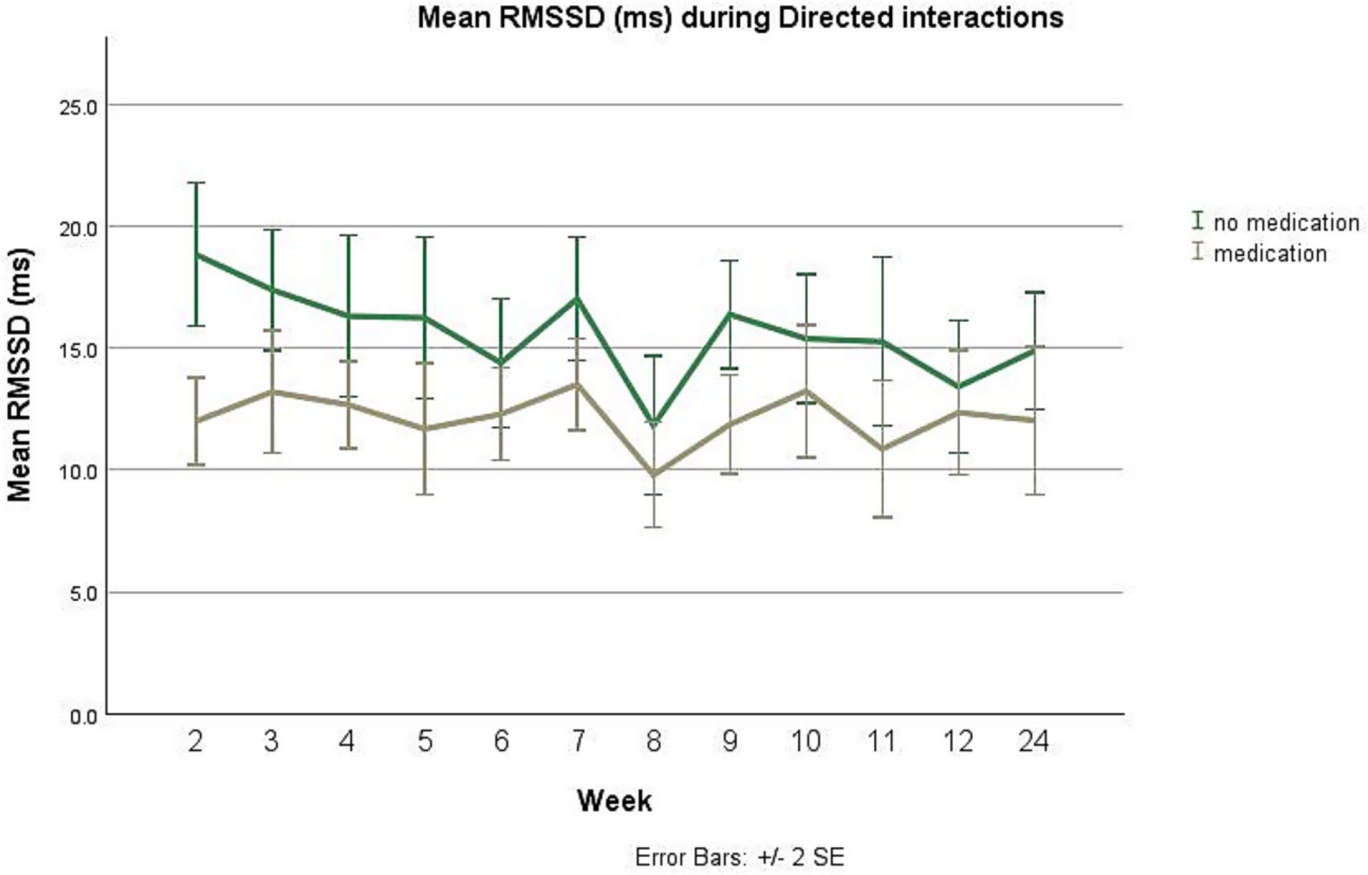
Heart rate variability (RMSSD), expressed in milliseconds (ms), by medication group during directed interactions with standard errors (SEs).

HRV (RMSSD) values during directed interactions were log_10_-transformed to improve the normality of the residuals. Model fit improved when fixed effects of group, number of deployments, experience with horses, sex, and interaction effects, with the exception of week * medication, were removed from the final model (first-order ante-dependence). During directed interactions, the log_10_-transformed RMSSD changed significantly over time (*F* (11, 70.85 = 4.95, *p* < 0.001, η^2^p = 0.435 indicating a large effect size with 43.5% of the variance explained). This temporal pattern was further qualified by a week * medication interaction effect (*F* (11, 70.85) = 2.12, *p* = 0.029, η^2^p = 0.248, indicating a large effect size with 24.8% of the variance explained) (Figure 8). *Post-hoc* comparisons revealed that across all time points, participants using medication generally exhibited lower log_10_-transformed RMSSD values than those not using medication (see Table 11 for model estimates); only the effect size changed over time. Inspection of temporal patterns indicated that log_10_-transformed RMSSD values were low in weeks involving the obstacle course (week 8) and riding activities (week 11). No main effects of medication were found (*F* (1, 59.70) = 3.49, *p* = 0.067).

**Table 11:** Estimated marginal means of heart rate variability (RMSSD), expressed in milliseconds (ms), by medication group during directed interactions with standard errors (SEs) and 95% confidence intervals (CIs).

Week	No medication				Medication			
	Model estimates	SE	95% CI (lower)	95% CI (upper)	Model estimates	SE	95% CI (lower)	95% CI (upper)
2	1.226	0.035	1.157	1.296	1.054	0.045	0.964	1.145
3	1.192	0.033	1.126	1.259	1.086	0.043	1.000	1.172
4	1.144	0.035	1.074	1.214	1.091	0.044	1.003	1.179
5	1.136	0.039	1.058	1.214	1.009	0.050	0.910	1.109
6	1.097	0.035	1.027	1.167	1.059	0.045	0.970	1.149
7	1.195	0.033	1.129	1.260	1.110	0.042	1.026	1.194
8	0.979	0.044	0.892	1.067	0.960	0.056	0.848	1.071
9	1.174	0.035	1.105	1.243	1.028	0.045	0.940	1.117
10	1.139	0.039	1.062	1.216	1.070	0.050	0.971	1.170
11	1.110	0.040	1.030	1.191	0.980	0.052	0.876	1.083
12	1.053	0.042	0.969	1.137	1.045	0.054	0.938	1.153
24	1.135	0.038	1.059	1.210	1.042	0.050	0.944	1.141

#### 3.5.3 Vector magnitude (counts per minute)

**Figure 9:**
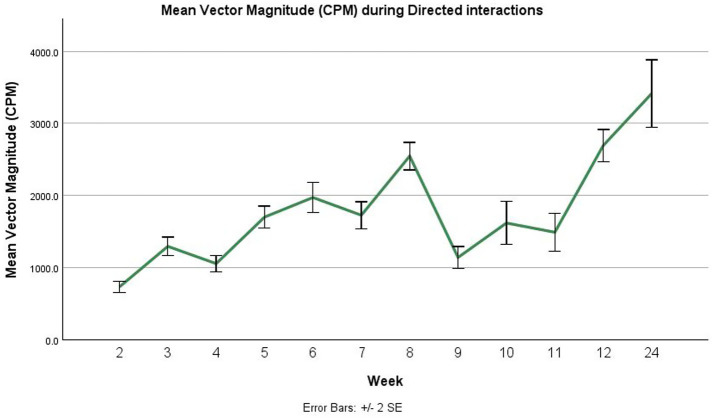
Vector magnitude (VM), expressed in counts per minute (CPM), during directed interactions with standard errors (SEs).

VM values during directed interactions were log_10_-transformed to improve the normality of the residuals. Model fit improved when fixed effects of number of deployments, experience with horses, medication, sex, and interaction effects were removed from the final model with a factor analytic first-order heterogeneous covariance structure. The final model showed a significant change in log_10_-transformed VM over time (*F* (11, 71.37) = 77.34, *p* < 0.001, η^2^p = 0.923 indicating a large effect size with 92.3% of the variance explained); see Figure 9 for raw values. This indicates that the activity did change over time during directed interactions. *Post-hoc* comparisons showed higher values were observed in weeks 8, 12, and 24 (see Table 12 for model estimates). Group significantly affected the log10-transformed VM values (*F* (8, 48.10) = 4.610, *p* < 0.001, η^2^p = 0.434 indicating a large effect size with 43.4% of the variance explained). *Post-hoc* comparisons indicated modest differences between groups, with group 9 showing the lowest values and group 4 the highest (see Table 13 for model estimates). However, confidence intervals largely overlapped across most groups, suggesting that differences were relatively small and not consistently distinct between all groups.

**Table 12:** Estimated marginal means of the vector magnitude (VM), expressed in counts per minute (CPM), by week during directed interactions with standard errors (SEs) and 95% confidence intervals (CIs).

Week	Model estimates	SE	95% CI (lower)	95% CI (upper)
2	2.836	0.025	2.787	2.885
3	3.087	0.020	3.047	3.127
4	2.992	0.024	2.944	3.039
5	3.213	0.018	3.176	3.249
6	3.262	0.026	3.210	3.314
7	3.194	0.031	3.133	3.255
8	3.395	0.018	3.360	3.431
9	3.012	0.030	2.953	3.071
10	3.119	0.036	3.048	3.191
11	3.088	0.036	3.016	3.159
12	3.415	0.022	3.372	3.458
24	3.501	0.043	3.413	3.589

**Table 13:** Estimated marginal means of the vector magnitude (VM), expressed in counts per minute (CPM), by group with standard errors (SEs) and 95% confidence intervals (CIs).

Group	Model estimates	SE	95% CI (lower)	95% CI (upper)
1	3.129	0.027	3.076	3.183
2	3.217	0.027	3.163	3.271
3	3.217	0.027	3.163	3.270
4	3.259	0.043	3.173	3.344
5	3.206	0.031	3.144	3.268
6	3.197	0.028	3.140	3.254
7	3.143	0.031	3.082	3.205
8	3.172	0.034	3.105	3.240
9	3.046	0.028	2.989	3.103

## 4 Discussion

To our knowledge, this is the first longitudinal study of EAIs in treatment-resistant military PTSD combining repeated psychophysiological measurements with long-term follow-up of up to 1 year, using a standardized protocol across multiple cohorts. During the EAI program in the current study, PTSD symptoms decreased, sleep quality improved, and quality of life increased significantly in military veterans with treatment-resistant PTSD, and these effects persisted after the program ended. In contrast, there were no indications that EAI is associated with structural or lasting physiological changes, as there were no significant differences in HR and HRV pre- and post-intervention when measured in veterans’ home environments. However, during EAI, HR decreased and HRV increased after the first week during free interactions with the horse, signalling adaptation to acute stress.

### 4.1 Psychometric questionnaires

The results of this study are in line with other studies that also demonstrated that EAI was associated with a significant decrease in PTSD symptoms (Arnon et al., 2020; Fisher et al., 2021; Lanning et al., 2017; Lanning et al., 2018; Malinowski et al., 2018; Romaniuk et al., 2018; Willmund et al., 2021; Zhu et al., 2021). After cessation of the program at the 3-month follow-up (week 24), PTSD symptoms remained low, comparable to findings from other studies with a 3-month follow-up (Arnon et al., 2020; Fisher et al., 2021; Romaniuk et al., 2018; Zhu et al., 2021). The raw PCL-5 scores indicate that the decrease in PTSD symptoms is clinically relevant (>10 points) in week 12 and approaches the 31-point cut-off score after the 1-year follow-up (Blanchard et al., 2023; Marx et al., 2022). This reflects sustained treatment effects and/or longer-term recovery processes, as some therapeutic benefits may have continued to consolidate after the intervention period, for example, through ongoing behavioural changes, increased engagement in daily activities, or continued application of coping strategies acquired during the intervention. Although spontaneous recovery over time cannot be ruled out, this is improbable since the study focused on treatment-resistant participants who had persistent symptoms despite prior treatment.

There is an association between sleep disturbances and PTSD (Harvey et al., 2003; Schoenfeld et al., 2012); both sleep quality and quantity are affected by PTSD (Stout et al., 2017). In military personnel who were treated for insomnia, reductions in PTSD and depression were also related to improvement in sleep quality (Rusch et al., 2015). This study found similar results, alongside a decrease in PTSD symptoms, an improvement in sleep quality was also found during EAI. Although sleep quality improved significantly, the population mean did not meet the criteria for “good sleepers” or “no PTSD.” This is in line with other studies on women with PTSD (Galovski et al., 2009; Porten et al., 2025) that found that TFTs significantly improved sleep quality, but the population did not reach “good sleeper” status. A meta-analysis reports a range of minimal clinically important difference (MCID) values of 1.14 to 1.80 points for the PSQI total score (Geng et al., 2025), which is comparable to the difference in our study at week 12. This indicates that the EAI program should be at least 12 weeks long.

The literature indicates that PTSD reduces quality of life (Dams et al., 2020; Schnurr et al., 2009), and similar EQ-VAS scores (61.0) are reported (Dams et al., 2021) compared with our study. Quality of life increased significantly in military veterans with treatment-resistant PTSD during EAI, which is in line with other studies that investigated the effects of cognitive behavioural therapy and pharmacotherapy treatment for PTSD (Le et al., 2013) and EAI (Arnon et al., 2020; Monroe et al., 2019; Romaniuk et al., 2018). It is unclear whether this is clinically relevant as the minimally important difference (MID) for the EQ-VAS ranged from 0.42 to 23.0, depending on baseline score, disease type, and treatment (Cheng et al., 2024). In this study, the effects were more pronounced in the latter weeks of the EAI program and persisted after its termination. This should be considered when developing EAI programs and determining their duration.

### 4.2 Physiological parameters

The literature reports inconsistent results on HRV indices of treatment efficacy in PTSD, although most studies found significant increases in HRV indices after treatment, which confirms the expectation that PTSD causes a dysfunctional autonomic nervous system that is normalized after treatment (Cao-Noya et al., 2025). This study did not find significant differences in HR and RMSSD when measured at home pre- and post-intervention, providing no evidence that EAI was associated with structural adaptations in the autonomic nervous system.

During baseline conditions at the riding school, HR was elevated in weeks 1 and 9, but no significant effect of week on RMSSD was found. HR reflects overall cardiac output (including both sympathetic and parasympathetic activity), as the RMSSD (reflecting parasympathetic tone) did not differ significantly; an increase in sympathetic tone could explain this. Interestingly, participants with prior horse experience had lower HRV, as evidenced by RMSSD. Although seemingly counterintuitive at first, this may reflect anticipation-related arousal.

During free interactions, HR was higher in weeks 1 and 9, while the RMSSD was lower than in other weeks, indicating a decrease in parasympathetic tone. There were no robust time effects on activity for those particular weeks, and the assignment was comparable each week, focusing on relaxation and affiliative contact with the horse, suggesting that this pattern likely reflects psychological rather than physical stress. It is hypothesized that during free interactions in weeks 1 and 9, veterans experienced psychological stress, and that they successfully adapted in the following weeks. Week 1 represented the first time they interact with the horse, and week 9 was the first week in which they started horseback riding during directed activities in the afternoon, to which they may have anticipated, which they may have anticipated. The results suggest that the first-time encounter with the horse and anticipation of riding activities may be stressful, and that veterans successfully adapted to this acute stress over successive weeks. This cannot be explained solely by adaptation to the environment, as this does not account for the effects observed in week 9. This is in line with a study by Dennis et al. (2016) that demonstrated that people with high PTSD symptom severity showed increased HR and decreased HRV after acute stress, suggesting that autonomic dysfunction primarily resides in intervals following acute stress. A pilot study in veterans with PTSD showed that treatment as usual did not significantly affect HRV or symptom reduction (Tan et al., 2011), whereas the addition of HRV biofeedback, during which individuals learned to control their breathing, significantly increased HRV and reduced PTSD symptoms (Tan et al., 2011). Taken together, these findings raise the possibility that EAI may not directly cause autonomic changes but rather provides a context in which individuals can experience and potentially learn to regulate acute physiological arousal.

During directed interactions, HR and activity were higher, and RMSSD was lower during weeks involving more activity, such as the obstacle course (week 8) and riding activities. These findings highlight the strong influence of physical activity on autonomic measures and indicate the importance of controlling for physical activity when measuring the effects of EAI through physiological parameters such as HR and RMSSD. This also indicates that the effects of specific assignments with horses, like our directed activities, differ from those of interactions focused on relaxation and positive social contact, like our free interactions. The effects of week on HR and RMSSD were moderated by medication use during directed interactions. Similar findings were observed during baseline conditions and free interactions. Generally, participants using medication exhibited higher HR and lower RMSSD, indicative of elevated stress and/or altered autonomic regulation, suggesting a heightened stress profile.

### 4.3 Strengths and limitations

The strength of this study is its relatively large sample size and the combination of both psychometric and physiological parameters. Additionally, the repeated EAI sessions in a 12-week program, using a standardized protocol, allowed us to follow individual veterans’ responses to the program over time. These findings may be particularly relevant for veterans with high levels of avoidance or residual symptoms after TFT, for whom experiential and embodied interventions may offer an alternative route to engagement. An important limitation of this study is the lack of a control group. A wait-list control group was maintained throughout the project period. However, only nine individuals were considered for wait-list participation, as they met admission criteria and did not have ongoing therapeutic interventions. Of those wait-list participants, only 7 had completed a PCL-5 questionnaire at weeks 1 and 12, as some were admitted to the program before the 12-week period had progressed. Most individuals could not be assigned to the control group because they were engaged in concurrent psychological treatments during the waiting period, or because they did not meet the intervention inclusion criteria. It is recommended that future studies use an active control or treatment-as-usual comparison design rather than a strict wait-list approach. Confounding factors among participants were minimized as much as possible by including only veterans without ongoing therapeutic interventions elsewhere. However, participants were allowed to continue current medication use, and this may have overshadowed potential intervention-related changes. This multi-component intervention does not allow us to determine which intervention components drove the psychometric outcomes. Elements such as peer social support, researcher attention, structured scheduling, outdoor activities, and/or interactions with the horse may have contributed. Physiological parameters were collected specifically during standardized individual horse–participant interactions to increase the relevance to the horse-related component. However, we cannot exclude other effects such as placebo effects. Therefore, future studies using controlled comparative designs are needed to isolate component-specific effects. The results are presented as differences in the population over the weeks, because we are aware that the change may not be solely due to the intervention. Another limitation is that participants were self-selected, which may lead to selection bias and reduce generalizability. The results of this study may not apply to all veterans but may be most applicable to those attracted to interventions involving animals.

## 5 Conclusion

EAI is associated with improvements in PTSD symptoms, sleep quality, and quality of life in military veterans with treatment-resistant PTSD who previously received conventional trauma-focused interventions. In addition, during free interactions within EAI sessions, a decrease in HR and an increase in HRV were observed after the first week. We hypothesize that this may be considered a physiological sign of successful adaptation of military veterans with PTSD to acute stress during free interactions with horses. EAI was not associated with any structural changes in autonomic regulation outside the interactions with the horse. We hypothesize that EAI is an experiential intervention that may aid adaptation during acute stress, which may be a possible working mechanism of EAI and warrants testing in future studies. This study demonstrates that it is feasible to measure both psychometric and non-invasive physiological parameters and that combining these measures provides additional information. EAI research warrants further investigation in controlled trials, with close attention to underlying mechanisms and optimal program length, before considering clinical implementation in military mental healthcare.

## Data Availability

The raw data supporting the conclusions of this article will be made available by the authors, without undue reservation.
