# Supplementary material for: Horsepower: equine-assisted interventions are associated with reduced PTSD symptoms, improved sleep quality, and quality of life in military veterans with treatment-resistant PTSD
Source: Front Psychol. 2026 Jun 9;17:1768001. doi: 10.3389/fpsyg.2026.1768001 (PMC13286912; doi:10.3389/fpsyg.2026.1768001)
Supplement: Supplementary file 1 [file Table_1.DOCX]

Table A: Non-significant fixed and interaction effects on PCL-5 scores from the linear mixed model, including F-values (df1, df2) and p-values.

| **Factor** | **F (df1, df2)** | **P-value** |
| --- | --- | --- |
| Group | F(8,41.65) = 1.67 | 0.134 |
| Gender | F(1,42.66) = 0.04 | 0.853 |
| Medication | F(1,42.97) = 1.52 | 0.225 |
| No. of deployments | F(1,42.60) = 0.51 | 0.480 |
| Week * Gender | F(5,73.13) = 0.58 | 0.713 |
| Week * Medication use | F(5,72.63) = 0.22 | 0.955 |
| Week * Experience horses | F(5,72.86) = 1.10 | 0.367 |
| Week * No. Deployments | F(5,73.08) = 0.82 | 0.538 |

Table B: Non-significant fixed and interaction effects on PSQI scores from the linear mixed model, including F-values (df1, df2) and p-values.

| **Factor** | **F-value (df1, df2)** | **P-value** |
| --- | --- | --- |
| Group | F(8,44.85) = 0.58 | 0.791 |
| Gender | F(1,46.49) = 0.18 | 0.676 |
| Medication | F(_1_,45.85) = 1.46 | 0.232 |
| Experience horses | F(_1_,46.23) = 3.54 | 0.066 |
| No. Deployments | F(1,46.68) = 0.53 | 0.470 |
| Week * Gender | F(4,59.60) = 1.07 | 0.378 |
| Wee * Medication use | F(4,55.81) = 2.33 | 0.068 |
| Week * Experience with horses | F(4,59.65) = 1.85 | 0.132 |
| Week * Number of deployments | F(4,56.06) = 2.24 | 0.077 |

Table C: Non-significant fixed and interaction effects on EQ-VAS scores from the linear mixed model, including F-values (df1, df2) and p-values.

| **Factor** | **F-value (df1, df2)** | **P-value** |
| --- | --- | --- |
| Group | F(8,44.98) = 1.36 | 0.242 |
| Gender | F(1,45.51) = 0.37 | 0.544 |
| Medication | F(1,45.53) = 0.11 | 0.739 |
| Experience horses | F(1,45.54) = 2.41 | 0.127 |
| No. Deployments | F(1,45.57) = 0.03 | 0.858 |
| Week * Gender | F(12,294.44) = 0.52 | 0.901 |
| Week * Medication use | F(12,289.72) = 0.48 | 0.926 |
| Week * Experience horses | F(12,288.36) = 1.00 | 0.447 |
| Week * No. Deployments | F(12,310.09) = 0.57 | 0.866 |

Table D: Non-significant fixed and interaction effects on heart rate (HR) pre-post intervention from the linear mixed model, including F-values (df1, df2) and p-values.

| **Factor** | **F-value (df1, df2)** | **P-value** |
| --- | --- | --- |
| Group | F(7,36.03) = 1.05 | 0.417 |
| Gender | F(1,36.50) = 0.05 | 0.820 |
| Medication | F(1,35.08) = 3.49 | 0.070 |
| Experience horses | F(1,34.53) = 0.06 | 0.808 |
| No. Deployments | F(1,34.24) = 0.05 | 0.818 |
| Week * Gender | F(12,40.12) = 0.94 | 0.339 |
| Week * Medication use | F(1,39.57) = 3.88 | 0.056 |
| Week * Experience horses | F(1,39.67) = 0.09 | 0.736 |
| Week * No. Deployments | F(1,38.88) = 0.39 | 0.537 |

Table E: Non-significant fixed and interaction effects on heart rate variability (RMSSD) pre-post intervention from the linear mixed model, including F-values (df1, df2) and p-values.

| **Factor** | **F-value (df1, df2)** | **P-value** |
| --- | --- | --- |
| Group | F(7,36.50) = 2.26 | 0.051 |
| Gender | F(1,37.26) = 0.66 | 0.423 |
| Experience horses | F(1,35.55) = 2.58 | 0.117 |
| No. Deployments | F(1,34.67) = 0.13 | 0.723 |
| Week * Gender | F(1,42.37) = 0.59 | 0.445 |
| Week * Medication use | F(1,40.96) = 2.40 | 0.129 |
| Week * Experience horses | F(1,41.44) = 0.89 | 0.351 |
| Week * No. Deployments | F(1,40.00) = 0.06 | 0.816 |
